# A comparison between manual and artificial intelligence–based automatic positioning in CT imaging for COVID-19 patients

**DOI:** 10.1007/s00330-020-07629-4

**Published:** 2021-03-19

**Authors:** Yadong Gang, Xiongfeng Chen, Huan Li, Hanlun Wang, Jianying Li, Ying Guo, Junjie Zeng, Qiang Hu, Jinxiang Hu, Haibo Xu

**Affiliations:** 1 Department of Radiology, Zhongnan Hospital of Wuhan University, Wuhan University, NO.169 Donghu Road, Wuchang District, Wuhan, 430071 Hubei Province People’s Republic of China; 2 Department of Radiology, Puren Hospital affiliated to Wuhan University of Science and Technology, NO.1 Benxi street, Jianshe 4th Road, Qingshan District, Wuhan, 430080 Hubei Province People’s Republic of China; 3 GE Healthcare, Computed Tomography Research Center, Beijing, 100176 People’s Republic of China

**Keywords:** Coronavirus, Artificial intelligence, Tomography, Radiation dosage

## Abstract

**Objective:**

To analyze and compare the imaging workflow, radiation dose, and image quality for COVID-19 patients examined using either the conventional manual positioning (MP) method or an AI-based automatic positioning (AP) method.

**Materials and methods:**

One hundred twenty-seven adult COVID-19 patients underwent chest CT scans on a CT scanner using the same scan protocol except with the manual positioning (MP group) for the initial scan and an AI-based automatic positioning method (AP group) for the follow-up scan. Radiation dose, patient positioning time, and off-center distance of the two groups were recorded and compared. Image noise and signal-to-noise ratio (SNR) were assessed by three experienced radiologists and were compared between the two groups.

**Results:**

The AP operation was successful for all patients in the AP group and reduced the total positioning time by 28% compared with the MP group. Compared with the MP group, the AP group had significantly less patient off-center distance (AP 1.56 cm ± 0.83 vs. MP 4.05 cm ± 2.40, *p* < 0.001) and higher proportion of positioning accuracy (AP 99% vs. MP 92%), resulting in 16% radiation dose reduction (AP 6.1 mSv ± 1.3 vs. MP 7.3 mSv ± 1.2, *p <* 0.001) and 9% image noise reduction in erector spinae and lower noise and higher SNR for lesions in the pulmonary peripheral areas.

**Conclusion:**

The AI-based automatic positioning and centering in CT imaging is a promising new technique for reducing radiation dose and optimizing imaging workflow and image quality in imaging the chest.

**Key Points:**

*• The AI-based automatic positioning (AP) operation was successful for all patients in our study.*

*• AP method reduced the total positioning time by 28% compared with the manual positioning (MP).*

*• AP method had less patient off-center distance and higher proportion of positioning accuracy than MP method, resulting in 16% radiation dose reduction and 9% image noise reduction in erector spinae.*

## Introduction

Accurate patient positioning and centering in computed tomography (CT) remains an important issue of concern for reducing dose and image noise [1–3]. One study reported that patients were mis-centered by 6 cm, resulting in up to 41% surface dose and 22% image noise increase [4]. To achieve high diagnostic image quality at reduced radiation dose, technologists make an extra effort to accurately select the anatomic scan range and carefully center the patients during CT scans. However, manual positioning and centering with accuracy is a time-consuming process and technologist-dependent and often inconsistent and non-optimal. For patients with infectious diseases, the interaction between technologists and patients also carries the potential cross-infection risk.

Recent advances in artificial intelligence (AI) technologies have demonstrated remarkable progress in recognizing and interpreting complex patterns in imaging data. The combination of AI and CT imaging can provide faster, more accurate, and efficient imaging-based diagnosis [5]. By virtue of 3D visual sensors, AI can identify the pose and shape of patients and realize an automated contactless image acquisition workflow. Yang Wang et al (2020) reported that U-HAPPY (United imaging Human Automatic Planbox for PulmonarY) CT has a function with automatic positioning and scanning, which helps to reduce the radiation dose [6]. Booij et al [7] and Saltybaeva et al [8] also reported the patient centering accuracy in CT using 3D cameras that relies on deep neural network for image contouring. Recently, GE Healthcare introduced a Revolution Maxima CT, which relies on deep learning algorithms and real-time depth-sensing technology to center patients, locate desired anatomies, and perform scan automatically. This CT scanner was successfully used for diagnosing COVID-19 patients in our hospital during the pandemic. However, applying AI to CT scanning technique is still at the exploratory stage. The purpose of this study was to analyze and compare the imaging workflow, patient positioning and centering accuracy, radiation dose, and image quality of COVID-19 patients who underwent several follow-up CT scans using the same CT protocol on a same CT machine but with either the conventional manual positioning (MP) mode or an AI-based automatic positioning (AP) mode. We hope our findings may provide useful information on the characteristic of intelligent CT tools and help radiologists to achieve better images at lower radiation dose more efficiently, while to reduce the potential risks of medical workers exposing to patients with infectious diseases during CT examination.

## Materials and methods

The research was approved by Medical Ethical Committee (Approved Number. 2020037). Our institutional review board waived written informed consents for this study, and got consent from patients.

### Patients and data source

All the patients in our study had been diagnosed of COVID-19 according to the guideline of 2019-nCoV (Fifth Trial Edition) issued by the National Health Commission of China [9]. A total of 127 patients (68 men and 59 women; mean age, 57.7 years; age range, 20–83 years) with confirmed SARS-CoV-2 were identified who had undergone at least two chest CT studies at Wuhan Leishenshan Hospital between Feb 12, 2020, and Apr 10, 2020 (see more details in Table 1). These patients underwent the first chest CT using the conventional manual positioning and centering method, and an AI-based automatic positioning and centering method in the follow-up CT examination. The patients in our study were limited to the ones without the need for life-supporting tubes and other equipment and could follow verbal command. The interval time between the two scans was 5–8 days. Based on the different positioning methods, patients were categorized into the conventional manual positioning (MP) group and AI-based automatic positioning (AP) group, and all CT images and clinical data between the two groups were compared.

**Table 1:** Demographics and baseline characteristics of 127 COVID-19 patients included in this study

Characteristics	Patients (*n* = 127)
Age (years)	
Mean ± SD (Range)	58 ± 12 (20–83)
Sex	
Male	68 (53.5%)
Female	59 (46.5%)
Ratio of male to female	1.15:1
Body mass index (BMI, kg/m^2^)	
Mean ± SD (Range)	24.3 ± 3.2 (17.4–33.1)

Continuous value was presented as mean ± standard deviation (SD)

### CT image acquisition and reconstruction

**Fig. 1:**
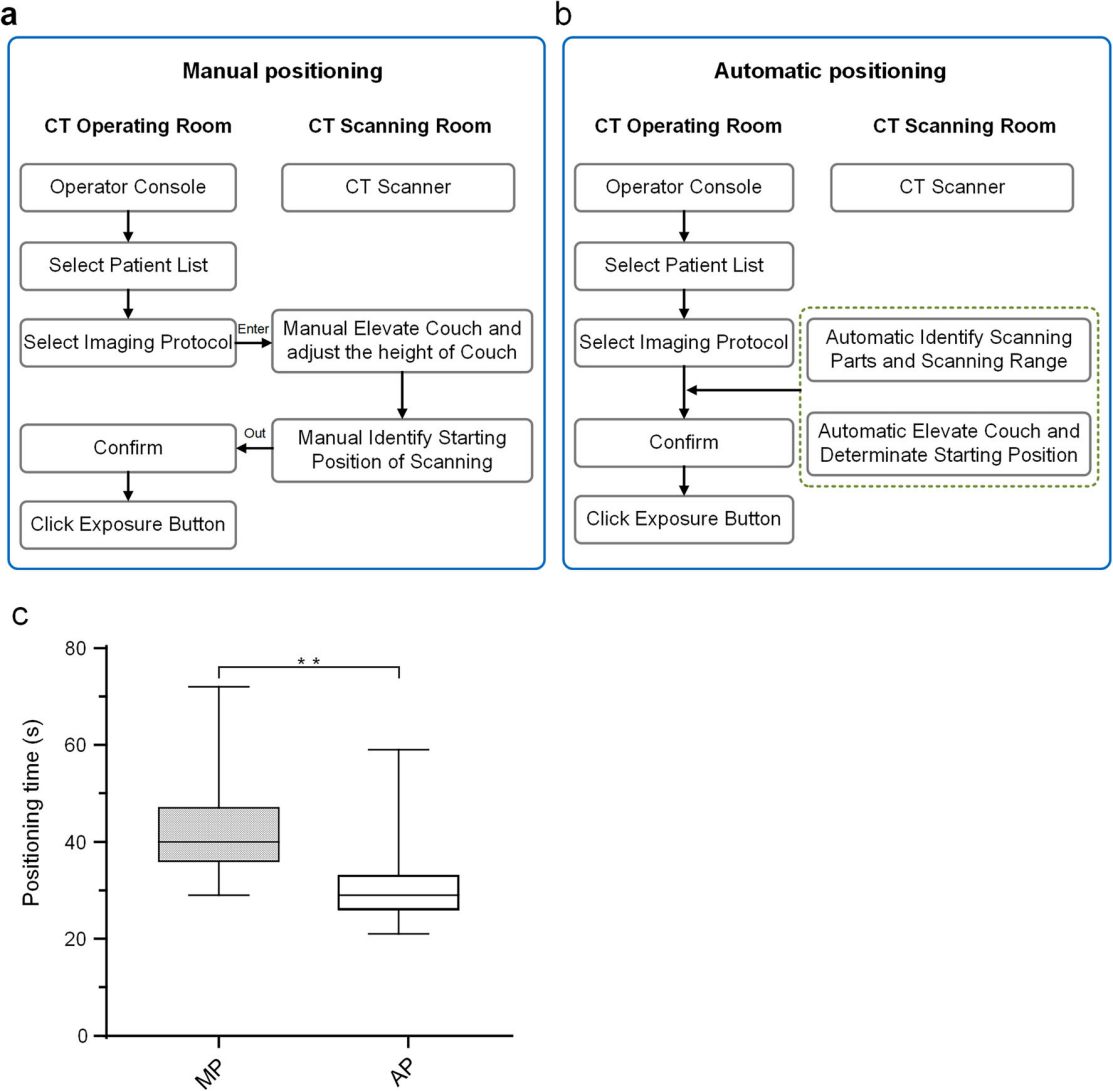
Schematic diagram for the operating steps of the manual positioning and automatic positioning. **a** Flowchart for the manual positioning. **b** Flowchart for the automatic positioning. **c** Quantification of positioning time. Statistical *p* value was calculated using a Wilcoxon signed-rank tests. In box plots, the central mark represents the median, and the edges of the box are the 25th and 75th percentiles. ** denote *p* < 0.01, *n* = 127 each

The imaging workflows for the MP and AP groups are shown in Fig. 1 a and b. The chest CT scanning was performed on a Revolution Maxima CT equipped with an AI-based automatic patient centering and anatomic positioning software (GE Healthcare) from the apex pulmonis to diaphragm. Both groups used the same scan protocol with the following parameters: tube voltage, 120 kVp; gantry rotation time, 0.4 s; pitch, 1.375:1; scan field-of-view (SFOV), 50 cm; slice thickness, 5 mm; tube current (mA), automated tube current modulation (ATCM) to obtain a noise index of 11.57; all axial images were reconstructed using a standard reconstruction algorithm with the standard kernel; reconstruction display field-of-view (DFOV), 35–50 cm; reconstruction thickness, 1.25 mm.

### AI-based automatic patient positioning and centering

**Fig. 2:**
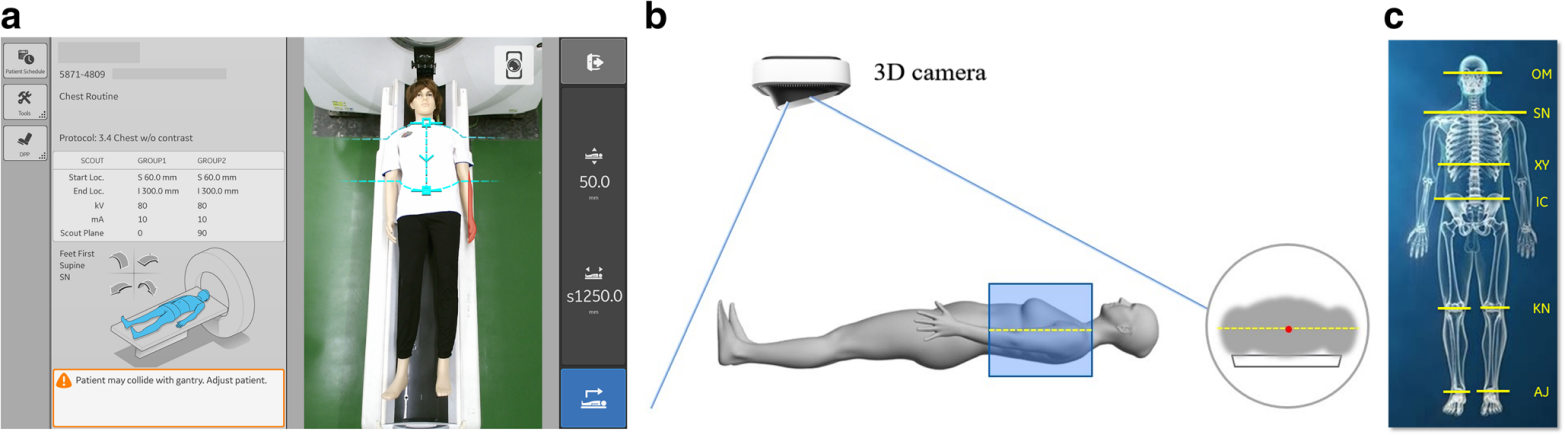
Schematic diagram of automatic positioning principle. **a** AI positioning uses a fixed, ceiling mounted, off the shelf, 2D/3D video camera that can determine distances to points in its field of view. When the user selects their desired protocol, the auto-positioning function uses the anatomical references and the scout range information to determine the landmark and the scan start and stop locations. **b** In the AI-based automatic patient positioning and centering technique, the 3D camera detects a depth information of patients and calculates the required table elevation through reading the dot pattern, capturing the infrared image to set the centering in the selected protocol and achieve accurate positioning. **c** The 8 supported anatomical references/landmarks

The AI-based automatic positioning uses a fixed, ceiling mounted, off the shelf, 2D/3D video camera that can determine distances to points in its field of view. It displays standard RGB video images on the CT system’s existing gantry-mounted touchscreens (Fig. 2 a, b). Information from the standard output of the camera is used, along with precise spatial information of the individual CT system’s gantry and table installation geometry, to determine the anatomical landmark location and the start and end locations for the scout scan(s). The scan protocol structure on the scanner contains a field for the anatomical reference. The 8 supported anatomical references for the automatic positioning method are orbital meatal (OM) baseline, sternoclavicular notch (SN), xyphoid (XY), iliac crest (IC), left and right knee (KN), and left and right ankle joint (AJ), as shown in Fig. 2 c. The automatic positioning software uses two deep learning algorithms (RGBLandmarkNet network and DepthLandmarkNet network) with different inputs that produce comparable outputs to identify all 8 of the anatomical landmarks on the patient’s body. All 8 of these identified landmarks are used to determine the patient orientation (head or feet first). In our study, the SN and IC landmarks were used for the chest scan. The RGBLandmarkNet network uses 2D video images as inputs and outputs all eight of the predefined landmark locations in X and Z. In parallel, the DepthLandmarkNet network uses the 3D depth data from the camera to also produce all eight of the predefined landmark locations. The 3D depth images are used to generate a “point cloud” on a mesh of points on the patient surface contour as determined from the depth information. The point cloud is then segmented to produce the body contour. The body contour is used to deterministically calculate the vertical geometric center of the patient. The center point location is then used to calculate the required table elevation for patient centering. With patient on the CT scanning table, the patient position and centering can be performed automatically with the one-touch button on the console in the control room.

### Assessment of image quality

**Fig. 3:**
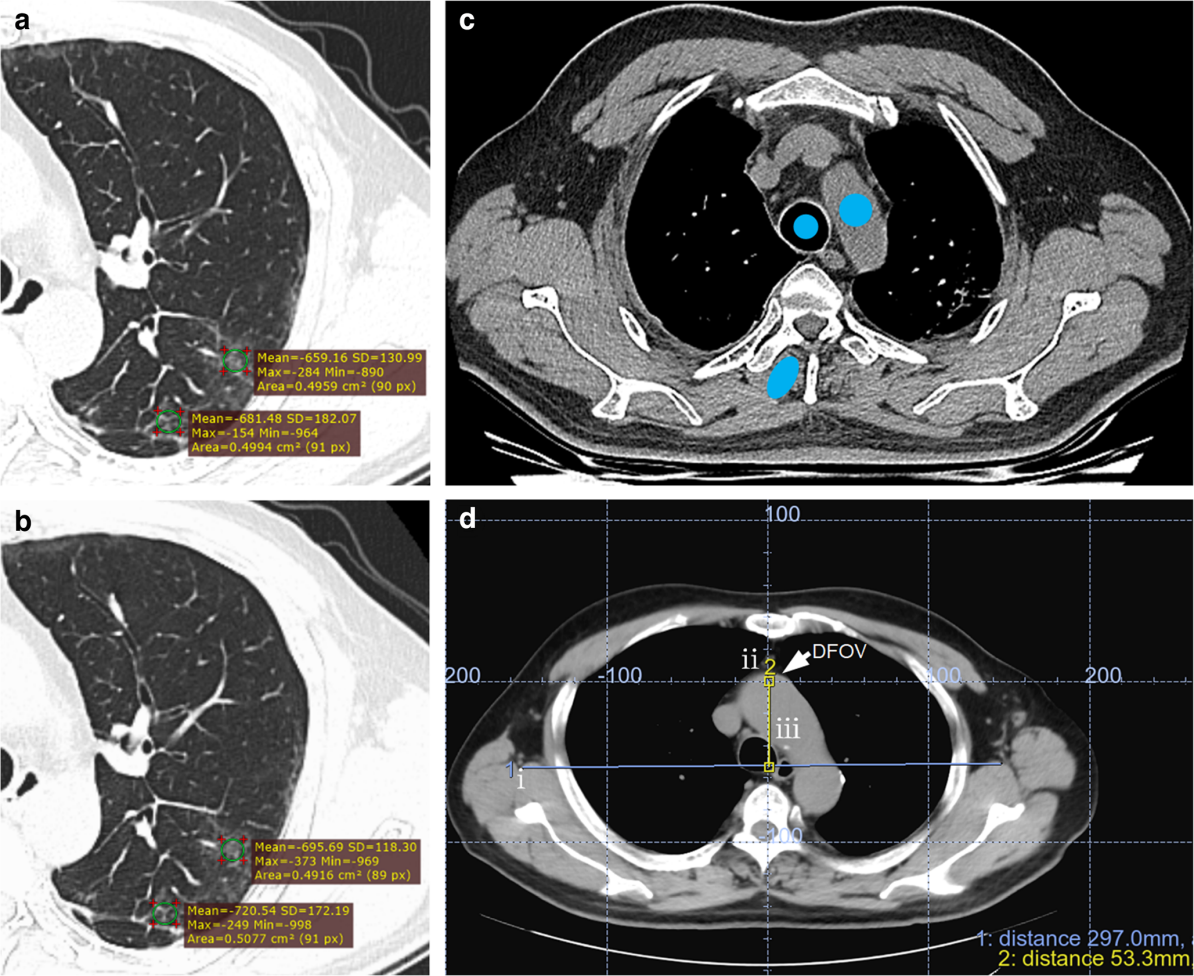
The measurement of CT value, noise and off-center distance on axial thin-section CT image in a COVID-19 patient. Lesions are shown along with ROI locations (green circles) used to acquire CT value measurements (mean ± SD) in different lung segments. The same patient went through two CT scanning with first with MP shown in **a** and second with AP after 6 days shown in **b**, demonstrating reduced lesion noise with AP method. **c** Axial CT slice of thorax is shown along with ROI locations used to acquire CT value measurements. The mean CT values and standard deviations (SDev) were calculated by drawing a 50 mm^2^ blue circular ROI in a homogeneous-appearing area of aorta, and trachea, and blue oval ROI in erector spinae in the chest area. **d** Measurement of off-center distance: (i) select a transverse image containing manubrium and draw a horizontal line that passes through both armpits, (ii) locate the center of the display field of view (DFOV) for the image by displaying the grid and selecting the center cross over point of the grid, and (iii) record the vertical distance from the center of DFOV to the horizontal line

The image quality was analyzed by three radiologists (H.B.X., J.X.H., Y.D.G) at a standard pulmonary display window setting (window level − 700 and window width 1500). The pulmonary lesions and the locations of ROI for these lesions were established by consensus. The mean CT value and standard deviation (SDev) in Hounsfield units (HU) of the aorta, trachea, and erector spinae in the upper and middle thorax areas were measured by placing a 50 mm^2^ region-of-interest (ROI) on a homogeneous-appearing area of these structures, as shown in Fig. 3 c. Three consecutive images were measured in each ROI area for each study, and the average value was determined. The mean and SDev of CT values within pulmonary lesions were also measured, as shown in Fig. 3 a, b. The pulmonary lesions mainly included ground glass opacification, consolidation opacification, and interstitial thickening. Other radiographic abnormality (hydrothorax, nodule or lump, cavitation or calcification, bronchiole or bronchiectasis, and emphysema) were also noted. The pulmonary segments were defined by referring to the branching patterns of bronchi [10–12]. If a lesion was located in the outer one-third of the lung, it was defined as peripheral; otherwise, it was defined as central. The signal-to-noise ratio (SNR) of the lesions was calculated based on the formula: *SNR* = Mean CT values/*SDev*. The image noise was represented using the SDev value.

### Positioning time

The positioning time was recorded by the CT technologist for each study. The positioning time was defined as the time from the patient lying on the CT examination bed to technologist finishing positioning and starting scanning.

### Off-center distance and positioning accuracy

**Fig. 4:**
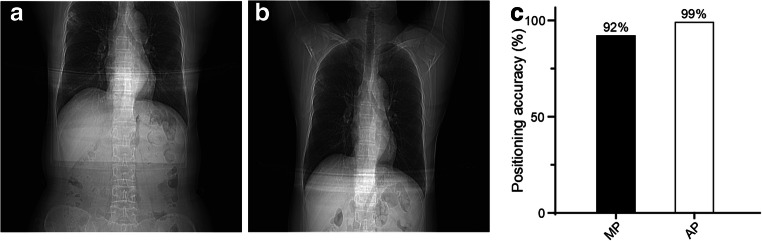
Comparison of positioning accuracy on chest CT topogram. **a** Inaccurate chest topogram, in which lung field was incompletely displayed. **b** Accurate chest CT topogram. **c** Comparison of positioning accuracy for chest topogram acquired by MP and AP (data were presented as *n* (%), where *n* was the number of patients with complete CT scout image; *n* = 127 each)

The patient off-center distance was measured using an axial CT image in the following steps: (i) select a transverse image containing manubrium and draw a horizontal line that passes through both armpits, (ii) locate the center of the display field of view (DFOV) for the image by displaying the grid and selecting the center cross over point of the grid, and (iii) record the vertical distance from the center of DFOV to the horizontal line (Fig. 3 d). For the positioning accuracy, a complete coverage should contain the apex pulmonis and diaphragm. Thus, if the images of apex pulmonis and diaphragm were fully covered, the patient positioning was considered successful; otherwise, it was defined incomplete or inaccurate (Fig. 4 a, b).

### Radiation dose

The volume CT dose index (CTDI_vol_ in mGy) and dose length product (DLP in mGy-cm) were recorded from the dose report image by the CT technologist for each study. The effective dose (ED in mSv) of the patient was calculated based on the formula: *ED* = *DLP* × *C*_*f*_, where the *C*_*f*_ represents the conversion factor for chest CT (*C*_*f*_ = 0.014 mSv/mGy-cm).

### Statistical analyses

Continuous variables were expressed as mean ± SD and compared using paired-sample *t* tests when the data were normally distributed; otherwise, the Wilcoxon signed-rank test was used; data distribution was tested with Shapiro-Wilk test. The categorical variables were expressed as number (percentage %) and compared with McNemar’s test. A two-tailed *p* value of less than 0.05 was considered statistically significant. All statistical analyses were conducted with IBM SPSS software (version 22.0).

## Results

### Baseline characteristics of objects

A total of 127 COVID-19 patients with a mean age of 57.7 years (ranged 20–83 years) were included in our study. Among them, there were 68 (53.5%) men and 59 (46.5%) women. The ratio of man to woman was 1.15:1. Their body mass index (BMI) values were in the range of 17.4–33.1 kg/m^2^, with an average value of 24.3 kg/m^2^ (see Table 1).

### Positioning time, accuracy, and off-center distance comparison

The positioning time in the AP group was significantly shorter than the MP group: 29.0 s ± 7.0 in AP vs. 40.0 s ± 11.0 in MP, *p* < 0.001 (Fig. 1 c). The positioning accuracy and off-center distance were determined using patient chest CT images. A significantly higher positioning accuracy was recorded in the AP group (126 of 127, 99.0%) than in the MP group (117 of 127, 92.0%) (Fig. 4). The patient off-center distances obtained with the AI-based method were all significantly less than those obtained with the manual method (mean off-center distance 1.56 cm ± 0.83 in the AP group vs. 4.05 cm ± 2.40 in the MP group, *p* < 0.001) (Table 2).

**Table 2:** Impact of positioning mode on radiation dose and off-center distance of COVID-19 patients

Positioning mode	CTDIvol (mGy)	DLP (mGy.cm)	ED (mSv)	Off-center distance (cm)	Scan range (cm)
MP (*n* = 127)	14.9 ± 2.3	523.4 ± 87.7	7.3 ± 1.2	4.05 ± 2.40	35.16 ± 3.32
AP (*n* = 127)	13.3 ± 2.4	437.4 ± 95.6	6.1 ± 1.3	1.56 ± 0.83	32.97 ± 3.89
*p* value	< 0.001	< 0.001	< 0.001	< 0.001	< 0.001

Data were presented as mean ± standard deviation (SD). *p* values denoted the comparison of different positioning mode groups. *MP*, manual positioning; *AP*, AI-based automatic positioning

### Radiation dose

The AI-based positioning group had significantly lower CTDIvoI value (13.3 mGy ± 2.4 vs. 14.9 mGy ± 2.3, *p <* 0.001), DLP value (437.4 mGy.cm ± 95.6 vs. 523.4 mGy.cm ± 87.7, *p <* 0.001), and ED value (6.1 mSv ± 1.3 vs. 7.3 mSv ± 1.2, *p <* 0.001) than the manual positioning group (Table 2).

### Image noise

The image noise was represented by the SDev measurement of the erector spinae in the upper and middle thorax areas. In both areas, the noise levels in CT images obtained with the AP method were all statistically lower than those in CT images obtained with the MP method: mean noise in the upper thorax, AP 49.7HU ± 7.3 vs. MP 54.1HU ± 9.3, and mean noise in the middle thorax, AP 48.9HU ± 8.5 vs. MP 53.9HU ± 9.1 (both *p* < 0.001) (Table 3). However, there was no significant difference for the noise values of the aorta and trachea in both groups.

**Table 3:** The influence of positioning mode on noise of chest CT images

Positioning mode	Upper thorax (HU)			Middle thorax (HU)		
	Aorta	Trachea	Erector spinae	Aorta	Trachea	Erector spinae
MP (*n* = 127)	38.7 ± 8.6	36.8 ± 7.3	54.1 ± 9.3	36.7 ± 7.7	37.3 ± 7.0	53.9 ± 9.1
AP (*n* = 127)	37.8 ± 10.1	35.7 ± 7.5	49.7 ± 7.3	35.6 ± 9.4	37.2 ± 8.1	48.9 ± 8.5
*p* value	0.344	0.120	< 0.001	0.316	0.913	< 0.001

Data were presented as mean ± standard deviation (SD). *p* values denoted the comparison of different positioning mode groups. *MP*, manual positioning; *AP*, AI-based automatic positioning

### Noise and SNR of pulmonary lesions

The pulmonary lesions could be found in any pulmonary segments in both groups. However, they predominantly distributed in the peripheral area of the lungs (766 of 791 lesions in the MP group vs. 927 of 957 lesions in the AP group) (Table 4). Overall, the AP group had marginally lower image noise and higher SNR for the lesions from the pulmonary segment point of view (Table 4). But for lesions located in the peripheral area, the AP group had significantly lower noise and higher SNR than the MP group (Table 5, Fig. 3).

**Table 4:** Noise and SNR from pulmonary lesions with MP and AP at different pulmonary segments

Location (segment)	All lesions (*n*, MP/AP)	Noise		SNC		*p* value	
		MP	AP	MP	AP	Noise	SNR
Upper lobe							
Apicale							
Right	45/55	171.0 ± 80.5	160.9 ± 75.3	4.9 ± 3.2	5.2 ± 3.1	0.522	0.508
Left	NA*	NA	NA	NA	NA	–	–
Posterius							
Right	35/47	157.2 ± 75.0	148.3 ± 67.3	5.2 ± 3.5	5.6 ± 3.4	0.582	0.589
Left	57/59	156.9 ± 71.4	132.8 ± 62.9	5.0 ± 3.1	5.6 ± 3.5	0.05	0.35
Anterius							
Right	46/55	149.1 ± 71.8	129.6 ± 62.7	5.0 ± 2.9	5.6 ± 2.9	0.153	0.248
Left	37/41	142.3 ± 55.7	125.8 ± 56.2	5.3 ± 2.5	6.4 ± 2.7	0.199	0.059
Middle lobe							
Mediale	29/41	144.7 ± 60.8	143.9 ± 78.3	5.5 ± 3.0	5.9 ± 3.2	0.962	0.595
Laterale	43/53	154.0 ± 67.3	135.8 ± 60.9	5.2 ± 3.5	5.8 ± 3.3	0.169	0.42
Lingulare							
Superius	27/31	124.9 ± 61.6	104.2 ± 45.6	5.3 ± 2.4	6.1 ± 2.6	0.149	0.258
Inferius	42/52	147.8 ± 70.7	130.1 ± 64.5	5.2 ± 2.8	6.2 ± 2.7	0.208	0.156
Lower lobe							
Superius							
Right	57/71	144.1 ± 59.9	141.7 ± 65.5	5.5 ± 3.8	6.0 ± 4.1	0.834	0.474
Left	49/59	148.8 ± 56.4	137.0 ± 55.1	5.1 ± 2.9	5.8 ± 3.0	0.277	0.224
Basale anterius							
Right	42/49	148.1 ± 69.1	130.8 ± 73.8	5.0 ± 2.8	5.2 ± 2.7	0.252	0.751
Left	46/52	149.7 ± 66.5	124.3 ± 60.6	5.3 ± 3.4	6.3 ± 3.0	0.051	0.15
Basale mediale							
Right	31/35	155.4 ± 64.0	152.8 ± 62.6	5.0 ± 3.0	5.4 ± 2.8	0.87	0.664
Left	NA*	NA	NA	NA	NA	–	–
Basale lateral							
Right	44/56	154.1 ± 65.0	143.6 ± 58.3	5.1 ± 2.9	5.4 ± 3.1	0.399	0.554
Left	51/63	164.5 ± 64.2	145.3 ± 54.0	4.5 ± 2.7	5.4 ± 3.0	0.086	0.129
Basale posterius							
Right	54/69	158.9 ± 62.2	146.9 ± 58.1	5.1 ± 2.7	5.5 ± 3.5	0.271	0.504
Left	56/69	146.4 ± 71.7	131.3 ± 71.6	5.4 ± 3.0	6.5 ± 3.6	0.242	0.072

*NA are presented as not applicable. Data are mean ± SD, where *n* is the amount of the pulmonary lesions with available data. *MP*, manual positioning; *AP*, AI-based automatic positioning; *SNR*, signal-to-noise ratio

**Table 5:** Distribution of noise and SNR with MP and AP of different lesion locations in chest CT

Lesion location	All lesions (*n*, MP/AP)	Noise		SNC		*p* value	
		MP	AP	MP	AP	Noise	SNC
Central	25/30	135.7 ± 54.1	132.6 ± 59.4	6.3 ± 2.7	6.6 ± 3.2	0.819	0.739
Peripheral	766/927	151.4 ± 66.9	137.5 ± 64.2	5.3 ± 3.3	6.1 ± 3.6	< 0.001	< 0.001

Data are mean ± SD, where *n* is the amount of the pulmonary lesions with available data. *MP*, manual positioning; *AP*, AI-based automatic positioning; *SNR*, signal-to-noise ratio

## Discussion

We analyzed and compared the imaging workflow, radiation dose, and image quality for COVID-19 patients examined using either the conventional manual positioning method or an AI-based automatic patient positioning and centering method. Our results indicated that the AI-based method not only automatically positioned patients with 99% accuracy and reduced the patient positioning time, but also reduced the radiation dose to patients and overall image noise by better centering the patients and with less positioning error margin.

Achieving high image quality at reduced radiation dose is always desirable. Reducing patient examination time and quickly diagnosing disease becomes even more necessary during the COVID-19 pandemic, because quickly screening and treating the patients should be the most critical measures for containing the pandemic. Introducing artificial intelligence into CT imaging provides us a new way to achieve it. The auto-positioning function automatically detects an anatomical landmark by deep learning algorithms and allows minimizing positioning actions into a single click operation, as illustrated in Figs. 1 and 2. This automatic positioning operation was approved to be efficient in our research, which plays an essential role in helping the response to the COVID-19 pandemic. Our results showed that the use of AI-based positioning for chest CT scanning resulted in a shorter time to complete the patient positioning. In particular, the chest positioning time was reduced in the AP group by 28%, as compared with the MP group. This automatic patient positioning operation was also approved to be accurate in our study. In our study, only one patient (1 out of 127) in the AP group required minor manual adjustment for the scan range after the AI selection. In addition, the scan range was more precise and was reduced by 6% overall based on the DLP report in the AP group which contributed to the additional 6% dose reduction for the patients in the AP group.

According to some related researches, off-center positioned patients substantially increase image noise and dose requirement [13]. In the AI-based automatic patient positioning and centering technique, the 3D camera detects a depth information of patients and calculates the required table elevation to set the centering in the selected protocol. The auto centering function optimizes the radiation dose and image quality without regard to operator’s skill. Our research found that the patient off-center distances with the manual positioning method were more than those with the AI-based method. In our study, the patient off-center distance was substantially reduced from the 4.05 cm ± 2.40 in the MP group to 1.56 cm ± 0.83 in the AP group. The off-center position reduction in the AP group subsequently reduced the radiation dose (CTDI) requirement to achieve similar image noise by 11%. Together with the tightened scan range brought about with the AI-based positioning, we achieved 16% dose reduction in the AP group (AP 6.1 mSv ± 1.3 vs. MP 7.3 mSv ± 1.2).

When patients are mis-positioned in the gantry, not only the radiation dose requirement is artificially increased, the image quality often underperforms as well [14]. Our results also indicated that the noise level in CT images obtained with AP mode, particularly in the erector spinae and the lesions in the peripheral lung regions, was statistically lower than those in CT images obtained with MP mode. Chung et al [15] reported that the lung lesions in COVID-19 patients are predominantly distributed in the peripheral region of the lungs. Hence, the centered patients with AP mode may have the positive impact on image quality of peripheral lesions and provide potential dose reduction opportunities.

Recently, Yang Wang et al reported the use of an intelligent system (U-HAPPY CT) [6] to reduce radiation exposure in chest CT application. Our results showed very similar radiation exposure reduction findings. Booij et al [7] and Saltybaeva et al [8] also reported the patient centering accuracy in CT using 3D cameras that relies on deep neural network for image contouring. Our results also agreed with their conclusions that the AI-based technique improved patient centering accuracy, and in turn improved image quality. In addition, we also demonstrated that the AI-based patient positioning technique on our CT scanner was able to position the patients automatically with one click of the button and no human contact that not only reduced positioning time, improved workflow but also minimize the potential cross-contamination between patients and medical workers, which is even more relevant during epidemic or pandemic such as COVID-19.

Our research had some limitations. Firstly, since this scanner was purchased specifically for combating the COVID-19 pandemic, the patients were limited to COVID-19 patients due to the safety requirement; our study may suffer from confounding bias due to the relatively small number of patients. But the AI-based positioning method should not be limited to COVID-19 patients, and more evaluation with larger number of patients is needed to generalize the conclusions. In addition, amidst the fear and confusion during pandemic, the scan protocols for the newly purchased CT scanner was not fully optimized and iterative reconstruction algorithms were not used because we were not sure how the iterative reconstruction algorithms would interact with the image quality and diagnosis of COVID-19 patients. Specifically, the radiation doses used for the COVID-19 patients were on the high end of the dose spectrum and left a lot of room for improvement. However, we believe the radiation dose should not affect the conclusions of our study. Secondly, we only evaluated one CT scanner from one manufacturer. Additional studies are needed to investigate the generality of AI positioning on different CT scanners. Thirdly, although the measurements in this study were performed on the same machine in the same patient, there were still differences in the status of the patient compliance during the two CT examinations.

In summary, our study indicates that the use of AI-based automatic patient positioning and centering results in less radiation dose, higher examination efficiency, higher positioning accuracy, and higher image quality in CT imaging the chest. This technique has important added clinical value for diagnosing infectious patients such as COVID-19 patients to reduce the cross-infection risks between patients and medical workers.

## Abbreviations

AI: Artificial intelligence
AP: AI-based automatic positioning
COVID-19: 2019 coronavirus disease
MP: Manual positioning
SNR: Signal-to-noise ratio
